# Whole Genome Transcriptomic Analysis of Ovary Granulosa Cells Revealed an Anti-Apoptosis Regulatory Gene DLGAP5 in Polycystic Ovary Syndrome

**DOI:** 10.3389/fendo.2022.781149

**Published:** 2022-03-18

**Authors:** Yan Deng, Hu Li, Yi Song, Juan Cen, Yuying Zhang, Yi Sui, Dexuan Cui, Tin Chiu Li, Yan Xu, Chi Chiu Wang, Pui Wah Jacqueline Chung, Tao Tang

**Affiliations:** ^1^Department of Obstetrics & Gynaecology, Faculty of Medicine, The Chinese University of Hong Kong, Shatin, Hong Kong; ^2^The First Affiliated Hospital, Jinan University, Guangzhou, China; ^3^Department of Gynecology, Pan Yu Central Hospital, Guangzhou, China; ^4^Cancer Institute of Panyu Central Hospital, Guangzhou, China; ^5^Key Laboratory of Natural Medicine and Immune Engineering, Henan University, Kaifeng, China; ^6^Department of Urology, the Sixth Affiliated Hospital of Guangzhou Medical University, Qingyuan People’s Hospital, Qingyuan, China; ^7^Department of Nutrition, The First Affiliated Hospital of Sun Yat-sen University, Guangzhou, China; ^8^School of Biomedical Science, The Chinese University of Hong Kong, Shatin, Hong Kong

**Keywords:** PCOS, transcriptomic analysis, ovary granulosa cells, DLGAP5, apoptosis

## Abstract

The mechanisms underlining pathogenesis of polycystic ovary syndrome (PCOS) remain largely unknown. Dysfunction of ovarian granulosa cells plays an important role. The present study performed the lncRNA and mRNA profiling by whole genome transcriptomic sequencing of ovary granulosa cells from women with PCOS and investigated the potential role of differentially expressed gens (DEGs) in the pathomechanism of PCOS. In total, 1,936 DEGs (30 upregulated and 1,906 downregulated mRNAs and lncRNAs) were identified in the ovary granulosa cells between control and PCOS group. Functional enrichment analysis showed that DEGs were mainly associated with cytokine–cytokine receptor interaction, neuroactive ligand–receptor interaction, and olfactory transduction. qRT-PCR validated the upregulation of DLGAP5 mRNA in ovary from PCOS group when compared to control group. Immunostaining and TUNEL assays showed that DLGAP5 protein level was increased while apoptosis was decreased in follicles of ovary in PCOS group. *In vitro* functional assays showed that DLGPA5 knockdown repressed viability and proliferation, but enhanced apoptosis and disrupted cell cycle in granulosa cells; while DLGAP5 overexpression had the opposite effects in granulosa cells. In conclusion, the study showed differentially expressed lncRNA and mRNA profile in the granulosa cells in ovaries of PCOS. Functional results demonstrated that DLGAP5 is a dysregulated candidate gene in the pathogenesis of PCOS, especially granulosa cell apoptosis and proliferation.

## Introduction

Polycystic ovary syndrome (PCOS), one of the common endocrine abnormalities, affects about 5–10% of women in reproductive age and contributes to 75% of anovulatory infertility. It is a complex, heterogeneous syndrome of uncertain etiology. The characteristics of PCOS include hyperthecosis, hyperinsulinemia, hyperandrogenemia, anovulation and polycystic ovaries (1–4). In recent years, increasing awareness of this syndrome in the medical communities and general population and women with PCOS are likely to develop metabolic syndrome and other comorbidities. Because of its heterogeneity of presentations, the definition of PCOS is always controversial in different clinical disciplines. Therefore, it is still a challenge for research scientists and clinical physicians to elucidate the origins and identify the pathological mechanism (5).

Granulosa cells provide a suitable microenvironment for follicular development and oocyte maturation. As a result, they are important in ovarian folliculogenesis. In response to pituitary gonadotropin secretion, granulosa cells regulate the expression of a variety of genes that encode the components of steroidogenic pathways involved in estrogen biosynthesis (6). A line of studies have showed that in women with PCOS, granulosa cell dysfunction may contribute to abnormal folliculogenesis, excess production of intraovarian androgens and/or increased circulating anti-Müllerian hormone levels, however, the underlying mechanisms remain to be elucidated (7, 8). Therefore, it is clinically and scientifically important to understand the mechanisms underlying the dysfunction of granulosa cells in women with PCOS.

Accumulating evidence suggested that long non-coding RNAs (lncRNAs) and messenger RNA (mRNAs) are essential regulators of cell physiological and pathological processes. Since mRNAs that encode proteins were directly functional while lncRNA regulates the transcription of mRNAs through coexpression manner. Therefore, the study of these RNAs variation may be a key to discover the etiology of PCOS. Recent studies demonstrated lncRNAs and mRNAs are differentially expressed in granulosa cells of women with PCOS (9–11). However, these studies were not using the granulosa cells isolated from PCOS ovarian tissues but follicular fluid (FF). For the FF collection, the ovaries were stimulated with recombinant follicle-stimulating hormone (FSH) after pretreatment with gonadotropin-releasing hormone agonist, which impact on the gene expression and functions of granulosa cells, so the findings may be biased.

In this study, we recruited women with PCOS and control subjects for ovary tissue collection and GC isolation by laser-capture microdissection and conducted RNA sequencing (RNA-seq) to explore the expressing profile of lncRNAs and mRNAs in ovary of PCOS. DLGAP5 was identified to be upregulated in PCOS. We hypothesized that DLGAP5 inhibits apoptosis of granulosa cells in the follicles and reduces atresia during folliculogenesis, therefore growing follicles are increased in ovary of PCOS.

## Material and Methods

### Patient Recruitment

For the PCOS group, the inclusion criteria are as follows: 1) women aged not in perimenopausal period; 2) BMI not obese; and 3) PCOS diagnosed by the revised Rotterdam Diagnostic criteria. As for the control group, normal ovary tissue was collected from women who were diagnosed with endometrial carcinoma or cervical cancer without ovarian involvement and underwent ovarian biopsy. Only healthy ovaries were used in the control group under pathological examination; any abnormal ovaries were excluded. This study was approved by the Institutional Review Board at the Chinese University of Hong Kong (CREC2016.663).

### Laser Capture Microdissection for Ovary Granulosa Cells

Ovaries were sectioned onto neutral glass slides and lightly counterstained using the HistoGene LCM Frozen Section Kit (Arcturus). Laser capture microdissection (Leica, LMD700) was performed on granulosa cells. Briefly, membrane slides with sections were placed in the holder of microscopy. The Eppendorf tubes with 20 µl Buffer RL (GenElute™ Single Cell RNA Purification Kit) on the cap were set up to the collector tray. The real-time captured image was displayed in the software. After customizing the device and adjusting the focus to appropriate magnification, the section of interest was defined by drawing the lines. The detached specimen was dropped to the cap of Eppendorf tubes by gravity and lysed in the Buffer RL. Total RNA was extracted from granulosa cells according to the protocols of the manufacturer.

### RNA-Sequencing Analysis

The quality of the RNA obtained, namely, the integrity and size distribution, was confirmed using the Agilent 2100 Bioanalyzer pico-RNA chip (Aglient, CA, USA). Before the construction of an RNA-seq library, rRNA was removed from the total RNA samples using the RiboMinus Eukaryote Kit (Qiagen, Hilden, Germen). The NEB Next Ultra Directional RNA Library Prep Kit for Illumina (NEB, MA, USA) was used according to the instructions of the manufacturer to create an RNA library for RNA-seq. The resulting RNA-seq library was quantified using an Agilent 2100 Bioanalyzer and was run on the HiSeq PE150 platform (Illumina, CA, USA) for paired-end 150 RNA sequencing. Subsequently, bioinformatics analysis was performed. Gene expression was quantified using RPKM (Reads Per Kilo bases per Million reads). RPKM also considers the effect of sequencing depth and gene length on reads count. Log2 (FC) was calculated by edgeR software. RNAs with log2 (FC) >2 and p <0.05 were considered upregulated in PCOS compared to control samples. Similarly, RNAs with log2 (FC) <−2 and p <0.05 were determined to be downregulated. It is considered that the genes with more than 15 reads in the sequencing results are expressed, while the genes below 15 reads are not expressed. When the read count of all replicates of the target gene in PCOS group is greater than 15, and all replicates of in control group is less than 15, this gene was a specifically expressed transcript. The alternative splicing (AS) events, namely, Retained Intron (RI), skipped exon (SE), alternative 5’ splice site (A5SS), alternative 3’ splice site (A3SS), and mutually exclusive exons (MXE) were identified by the TAPIS pipelines. The differential AS events were detected using the program rMATS based on the RNA-seq data.

For Gene Ontology (GO) enrichment analysis, predicted target genes were mapped to GO terms in the Gene Ontology database. For Kyoto Encyclopedia of Genes and Genomes (KEGG) pathway enrichment analysis, predicted miRNAs target genes were mapped to KEGG annotation in the KEGG database.

### qRT-PCR

Approximately 100 ng total RNA were extracted from granulosa cells and reverse transcribed to cDNA using the commercial protocol. Quantitative Real-time Polymerase Chain Reaction was performed by using Power SYBR^®^ Green PCR Master Mix (Applied Biosystems, Foster City, California). The relative expression of target RNA was determined by dividing the target amount by endogenous control amount to obtain a normalized target value. Then the normalized values of the target RNA were compared among the samples.

### Immunohistochemistry

Formalin-fixed paraffin-embedded sections (5 μm) sections were deparaffinized in three Xylene baths and rehydrated in graded ethanol concentrations. Antigen retrieval was performed with citric acid antigen retrieval buffer (PH 6) in a microwave oven for 8 min. Endogenous peroxidase was blocked with 3% hydrogen peroxide in phosphate-buffered saline (PBS) for 25 min. Sections were then quenched with protein blocking buffer for 30 min followed by overnight incubation with DLGAP5 antibody (Abcam, Cambridge, United Kingdom). After rinsing, the sections were incubated with secondary antibody for 50 min. The staining was visualized with 3, 3’-diaminobenzidine (DAB) as chromogen and slides were counterstained with hematoxylin, dehydrated and finally mounted.

### Human Granulosa-Like Tumor Cell (KGN) Culture and Transfection

KGN cells were cultured in the DMEM/F12 medium supplemented with 10% fetal bovine serum (Gibco, Thermo Fisher Scientific, USA) in a humidified incubator containing 5% CO_2_ at 37 °C. DLGAP5 siRNA and non-sense siRNA (NC) were purchased from RioboBio (Guangzhou, China). SiRNAs were transfected using Lipofectamine 2000 (Invitrogen, USA) following the instructions of the manufacturer. Lentivirus LV-DLGAP5-EGFP expression vector with empty vector were provided by Cyagen Biosciences (Jiangsu, China). KGN cells at 40–50% confluence in 96-well plate were transfected with the Lentivirus vector according to the protocol of the manufacturer.

### CCK-8 Cell Viability Assay

CCK-8 assay kit (Beyotime Institute of Biotechnology, Shanghai, China) was used to assess the cell viability following the instructions of the manufacturer. After transfection for 24, 48, and 72 h, 10 μl of the CCK-8 solution was added to each well of the plate. One hour incubation later, the absorbance at 450 nm was measured.

### Terminal Deoxynucleotidyl Transferase dUTP Nick End Labeling (TUNEL) Assay

Ovarian tissue sections from both control group and PCOS group were examined for the presence of apoptotic cells using the DAB (SA-HRP) TUNEL Cell Apoptosis Detection Kit (Servicebio, Wuhan, China) according to the protocol of the manufacturer. For *in vitro* study, TUNEL assay was performed using the *In Situ* Cell Death Detection Kit (Roche, Merck, USA) and TUNEL BrightRed Apoptosis Detection Kit (Vazyme, Nanjing China, for cells transfected with LV-GFP) after transfection for 48 h. Images were captured using a microscope/fluorescent microscope (Eclipse Ti, Nikon, Melville, NY, USA).

### EdU Staining Assay

Cell proliferation was assessed using the EdU staining assay (Beyotime, Shanghai, China) after transfection for 48 h. EdU solution was added to cells and incubated for 2 h. Subsequently, fixative solution was added and incubate for 15 min, followed by Click reaction buffer incubation for 30 min and nuclei staining with 4’,6-diamidino-2-phenylindole (DAPI; Beyotime, Shanghai, China) for 5 min. Fluorescent images were captured using a fluorescent microscope. EdU positive rates were calculated as: (number of positive cells)/(total number of cells) × 100%.

### Apoptosis Assay

Apoptosis was evaluated using Annexin V-FITC and PI Apoptosis Detection Kit (KeyGEN BioTECH, Nanjing, China) according to the instruction of the manufacturer. In brief, after transfection for 48 h, cells were collected, suspended in binding buffer, and stained with Annexin V-FITC and PI for 15 min at room temperature. The rate of apoptosis was detected by flow cytometer (BD FACSCalibur™, USA)

### Cell Cycle Analysis

After transfection for 48 h, cells were harvested, fixed with 75% ethanol at −20°C overnight, and stained with propidium iodide (PI) solution with RNase A (KeyGEN BioTECH, Nanjing, China) for 60 min. Cell-cycle analysis was performed by flow cytometry (BD FACSCalibur™, USA).

### Western Blot Assay

Protein was extracted from transfected cells and 30 ug proteins were separated by 10% SDS-PAGE and transferred to PVDF membranes (Amersham; GE Healthcare, Buckinghamshire, England). After blocking with 1.5% skimmed milk at room temperature for 1 h, the membranes were incubated with DLGAP5, cleaved caspase-3, cleaved caspase-8, caspase-9, CDK2, cyclin C and cyclin D antibodies, and GAPDH antibody (ProteinTech, Chicago, IL, USA) at 4°C for overnight, followed by washing and incubation with horseradish peroxidase-conjugated secondary antibodies (CST, Massachusetts, USA) at room temperature for 1 h, respectively. The bands were visualized by an enhanced chemiluminescence system (Thermo Fisher Scientific, Rockford, IL, USA).

### Statistical Analysis

Data are presented as the mean ± SEM. Statistical comparisons between two groups were made by the Student’s t-test. *P <*0.05 was considered to be statistically significant.

## Results

### Clinical Characteristics of PCOS Patients and Control Subjects

A total of 19 samples (11 from PCOS and 8 from control) were collected for RNA-seq analysis. There were no significant differences in BMI, fasting glucose, cholesterol, HDL-cholesterol, triglycerides, and LDL- cholesterol between PCOS and control group. The average age for the PCOS group is 34.7 ± 2.9 years old, which is smaller than that for the control group (47.2 ± 5.8 years old; **Table 1**).

**Table 1:** Clinical characteristics of PCOS patients and control subjects.

Index	PCOS (n = 11)	Control (n = 8)	P-value
Age (y)	34.7 ± 2.9	47.2 ± 5.8	1.17E−06
BMI (kg/m^2^)	26.3 ± 4.0	24.2 ± 2.9	0.218
Fasting glucose (mmol/L)	5.7 ± 1.0	5.5 ± 1.6	0.700
Cholesterol (mmol/L_	4.8 ± 0.9	5.0 ± 0.6	0.726
HDL-Cholesterol (mmol/L)	1.1 ± 0.3	1.3 ± 0.3	0.329
Triglycerides (mmol/L)	1.9 ± 1.0	1.2 ± 0.4	0.118
LDL-Cholesterol (mmol/L)	2.8 ± 0.8	3.1 ± 0.6	0.483

### Quantification of Gene Expression

**Figure 1:**
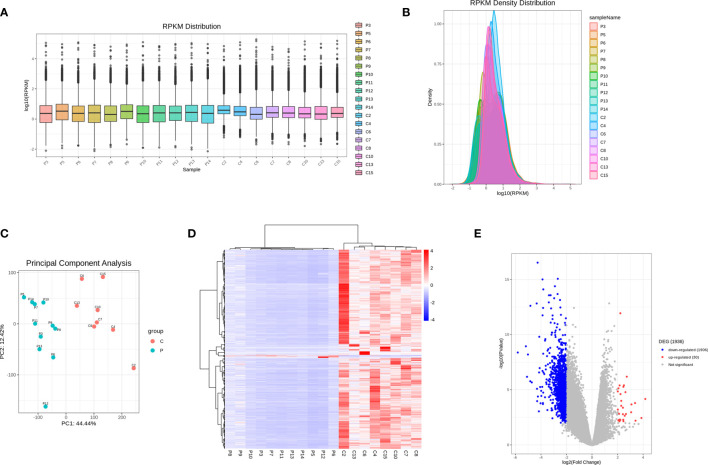
RNA-seq analysis of differentially expressed genes between PCOS and control group. **(A)** Boxplots of abundance of transcripts in each sample. **(B)** RPKM density distribution in each sample. **(C)** PCA of averaged, rank-normalized read counts from RNA-seq. **(D)** Heatmap analysis of differentially expressed genes between control group and PCOS group. **(E)** Volcano plots showing the significance and log2 fold-change (logFC) for all gene transcripts reliably detected in the RNA-seq analysis between control group and PCOS group.

As shown in **Figures 1A–C**, the overall gene expression levels of all samples in the control group and in the PCOS group were comparable, with no significant differences. PCA analysis using the RPKM values showed grouping information for each sample under different experimental conditions. Hierarchical clustering analysis of differentially expressed genes (DEGs) is shown in **Figure 1D**. The volcano plot results identified a total of 1,936 DEGs (30 upregulated and 1,906 downregulated, namely, 669 mRNAs, 11snoRNA, 1,195 lncRNAs, and 61 transcribed pseudogene) (**Figure 1E** and **Supplementary Data**).

### GO and KEGG Pathway Enrichment Analysis

**Figure 2:**
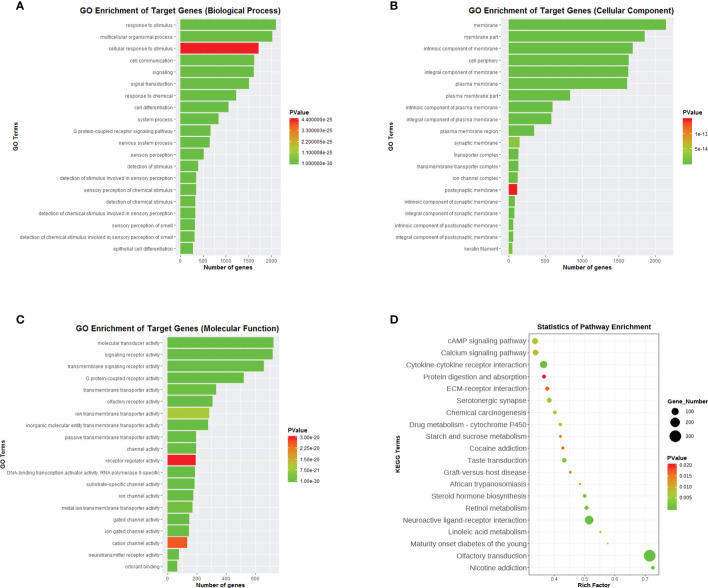
Functional enrichment analysis of differentially expressed genes. **(A)** GO enrichment analysis of DEGs in biological process. **(B)** GO enrichment analysis of DEGs in cellular component. **(C)** GO enrichment analysis of DEGs in molecular function. **(D)** KEGG enrichment analysis of DEGs. DEGs, differentially expressed genes.

To elucidate the possible functional significance of the observed changes in transcripts between PCOS and control groups, GO and KEGG analysis were performed. As shown in **Figure 2**, differentially expressed transcripts had GO annotations to biological processes, cellular components, and molecular functions. For biological processes, response to stimulus, cell communication, signaling and signal transduction process were significantly enriched. For KEGG analysis, cytokine–cytokine receptor interaction, neuroactive ligand–receptor interaction and olfactory transduction were significantly enriched (**Figure 2**).

### Alternative Splicing Analysis

**Figure 3:**
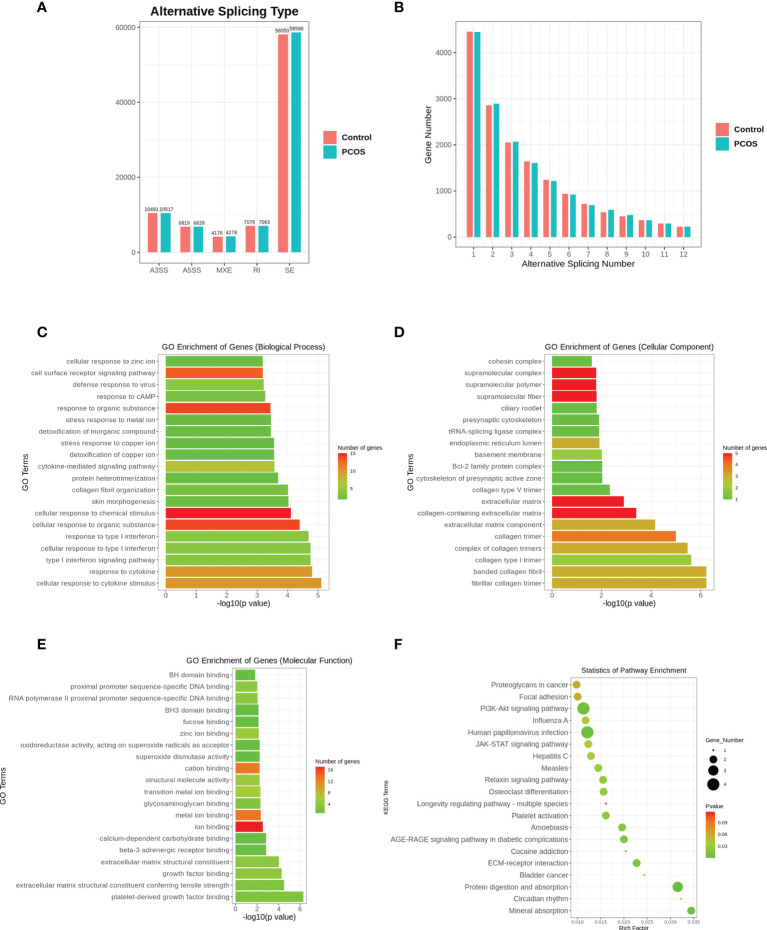
Alternative splicing analysis. **(A)** Alternative splicing types. A3SS, alternative 3’ splice site; A5SS, alternative 5’ splice site; MXE, mutually exclusive exons; RI, Retained Intron; SE, skipped exon. **(B)** Alternative splicing number. **(C)** GO enrichment analysis of alternative spliced genes in cellular component. **(D)** GO enrichment analysis of alternative spliced genes in molecular function. **(E)** GO enrichment analysis of alternative spliced genes in molecular function. **(F)** KEGG enrichment analysis of alternative spliced genes.

Alternative splicing events were detected in both PCOS and control groups. The number of events was similar in these two groups. Among the five most common types of AS events, skipped exon accounted for the highest proportion. We performed GO and KEGG enrichment analysis to investigate the biological functions associated with the differentially expressed genes (also differentially spliced genes) in which alternative splicing events occurred. Similarly, differentially expressed spliced genes had GO annotations to biological processes, cellular components, and molecular functions, with the most annotations to biological processes. KEGG pathway enrichment analysis indicated that PI3K-Akt signaling pathway, HPV infection, and protein digestion and absorption pathways were enriched among these alternatively spliced genes (**Figure 3**).

### Validation of the Selected Transcripts by qRT-PCR

**Figure 4:**
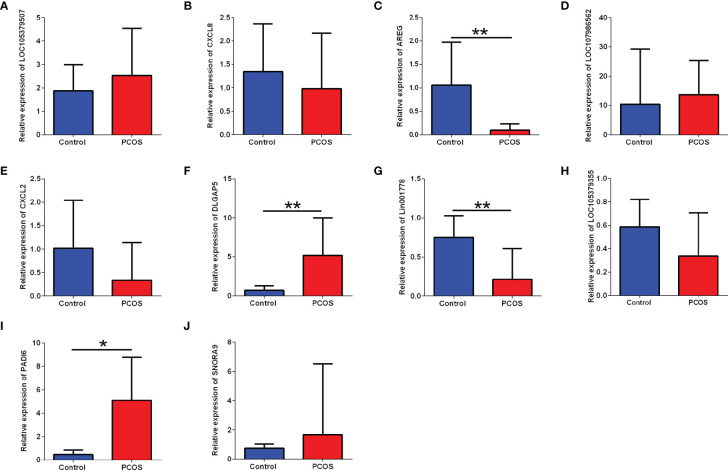
Validation analysis of selected differentially expressed candidate genes between control group and PCOS group. The mRNA expression levels of **(A)** LOC105379507, **(B)** CXCL8, **(C)** AREG, **(D)** LOC107986562, **(E)** CXCL2, **(F)** DLGAP5, **(G)** Lin001778, **(H)** LOC105379355, **(I)** PADI6, and **(J)** SNRA9 in the ovarian tissues between control group and PCOS group. N = 8–11. *P < 0.05 and **P < 0.01.

To verify the differentially expressed RNAs in the ovaries of PCOS, qRT-PCR was performed for the quantification of the expression of LOC105379507, CXCL8, AREG, LOC107986562, CXCL2, DLGAP5, Lin001778, LOC105379355, PADI6 and SNORA9. LOC105379507, CXCL8, AREG, LOC107986562, CXCL2 and Lin001778 were top downregulated mRNA and lncRNA. DLGAP5, LOC105379355, PADI6 and SNORA9 were top specifically expressed transcript. Among which, AREG and Lin001778 were significantly downregulated in the PCOS group when compared with the control group. DLGAP5 and PADI6 were upregulated in the PCOS group when compared to that in the control group (**Figure 4**). As DLGAP5 was the top one specifically expressed transcript, it was selected for further functional study. The ceRNA network analysis further revealed that DLGAP5 was extensively interacted with other non-coding RNAs (see **Supplementary Figure S1**).

### Histology Analysis of Ovarian Tissues

**Figure 5:**
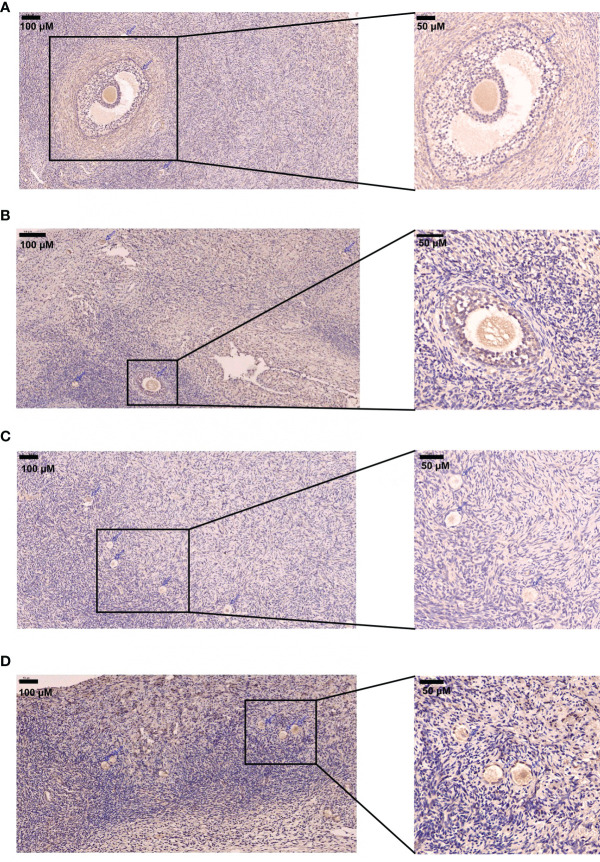
Immunostaining analysis of DLGAP5 in the ovarian tissues. **(A)** Immunostaining of DLGAP5 in control group. Scale bar = 200 µm. **(B)** Immunostaining of DLGAP5 in PCOS group. Scale bar = 200 µm. **(C)** Immunostaining of DLGAP5 in control group. Scale bar = 200 µm. **(D)** Immunostaining of DLGAP5 in PCOS group. Scale bar = 200 µm. Blue arrows indicate primary or antral follicles.

**Figure 6:**
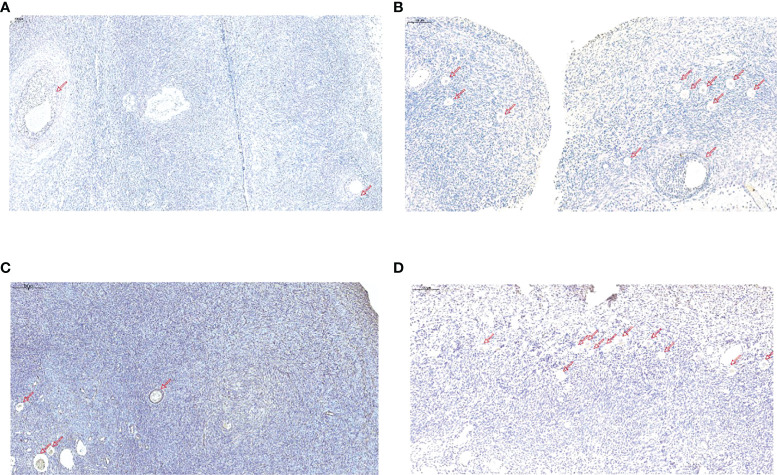
TUNEL analysis of ovarian tissues in the normal group and PCOS group. **(A, C)** TUNEL staining of ovarian tissues in control group. Scale bar = 200 µm. **(B, D)** TUNEL staining of ovarian tissues in PCOS group. Scale bar = 200 µm. Scale bar = 200 µm. Red arrows indicate primary, secondary or antral follicles.

The immunostaining was performed on the ovarian tissues. The immunostaining of DLGAP5 was examined in the ovarian tissues from the PCOS and control group. As shown in **Figure 5**, the DLGAP5 staining intensity of granulosa cells around the follicles in the PCOS group was slightly strong than that in the control group. The TUNEL staining results further demonstrated that the number of apoptotic cells in the ovarian group from the PCOS group was slightly decreased when compared to that from the control group (**Figure 6**).

### Effects of DLGAP5 Knockdown on the Granulosa Cell Proliferation and Apoptosis

**Figure 7:**
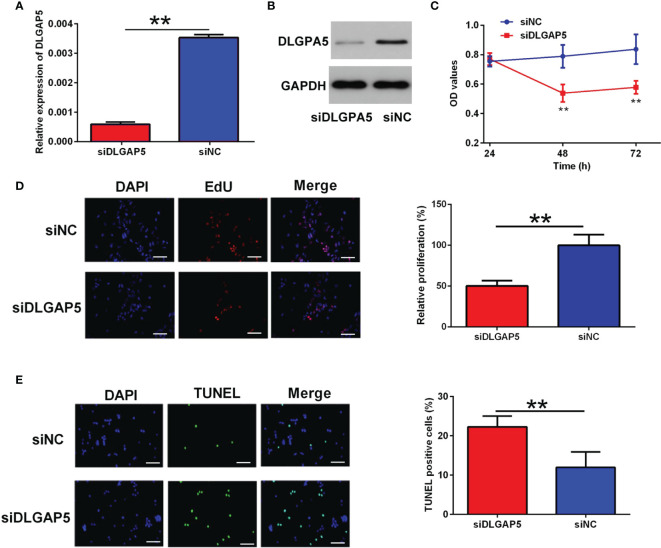
Effects of DLGAP5 knockdown on the granulosa cell proliferation and apoptosis. Granulosa cells were transfected with si-NC or si-DLGAP5, and after siRNA transfection, **(A)** the mRNA expression level of DLGAP5 in the granulosa cells was determined by qRT-PCR; **(B)** the protein expression level of DLGAP5 in the granulosa cells was determined by western blot; **(C)** the cell viability of granulosa cells was determined by CCK-8 assay; **(D)** the cell proliferation of granulosa cells was determined by EdU assay, scale bar = 50 µm; **(E)** the cell apoptosis of granulosa cells was determined by TUNEL assay; scale bar = 50 µm. N = 3. **P < 0.01.

DLGAP5 siRNA transfection caused a significant decrease in the mRNA and protein expression level of DLGAP5 in granulosa cells (**Figures 7A, B**). The CCK-8 assay showed that DLGAP5 knockdown significantly decreased the cell viability of granulosa cells (**Figure 7C**). The EdU assay showed that DLGAP5 silence significantly decreased the number of EdU-positive granulosa cells (**Figure 7D**). Consistently, the TUNEL assay showed that DLGAP5 knockdown markedly increased the number of TUNEL-positive granulosa cells (**Figure 7E**).

**Figure 8:**
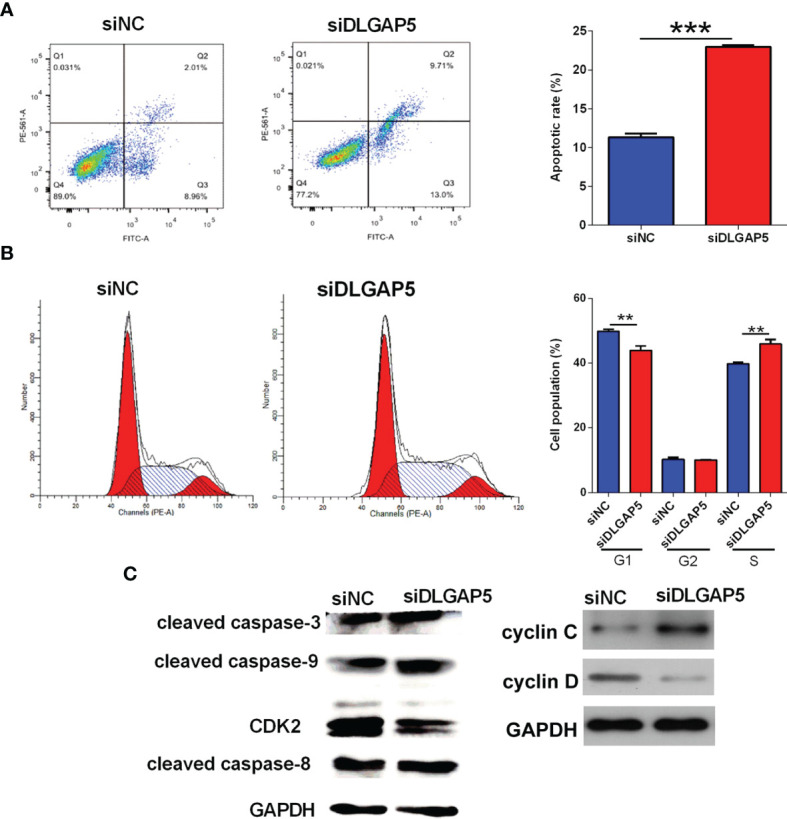
Effects of DLGAP5 knockdown on the granulosa cell apoptosis and cell cycle. Granulosa cells were transfected with si-NC or si-DLGAP5, and after siRNA transfection, **(A)** the cell apoptotic rates and **(B)** cell cycle of granulosa cells were determined by flow cytometry; **(C)** the protein levels of cleaved caspase-3, cleaved caspase-9, CDK2, cleaved caspase-8, cyclin C and cyclin D in granulosa cells were determined by Western blot assay. N = 3. **P < 0.01 and ***P < 0.001.

The flow cytometry was performed to determine the cell apoptosis and cell cycle of granulosa cells. As shown in **Figure 8A**, DLGAP5 knockdown significantly increased the number of apoptotic granulosa cells. The analysis of cell cycle revealed that DLGAP5 silence caused a significant decreased in the cell population of G1 phase and a significant increase in the S phase but had no effect on the cell population in the G2 phase (**Figure 8B**). The western blot results showed that DLGAP5 silence increased the protein levels of cleaved caspase-3, -8 (slightly) and -9 and cyclin C, but decreased the protein level of CDK-2 and cyclin D in granulosa cells (**Figure 8C**).

### Effects of DLGAP5 Overexpression on the Granulosa Cell Proliferation and Apoptosis

**Figure 9:**
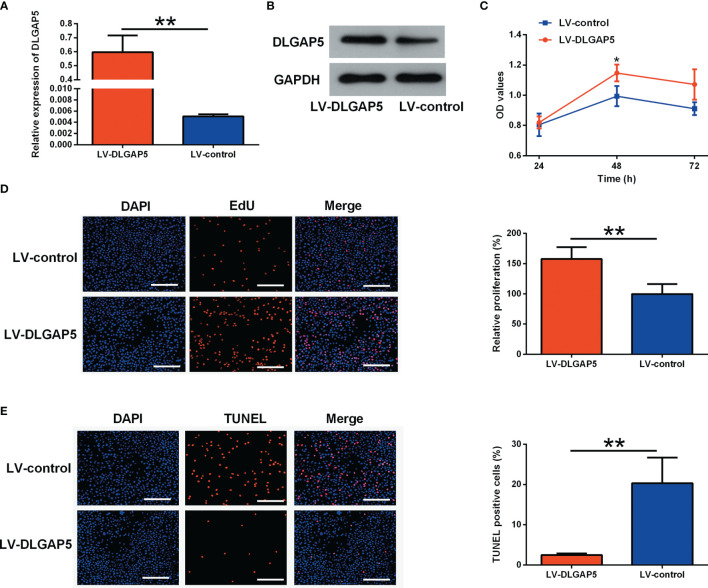
Effects of DLGAP5 overexpression on the granulosa cell proliferation and apoptosis. Granulosa cells were transfected with LV-control or LV-DLGAP5, and after transfection, **(A)** the mRNA expression level of DLGAP5 in the granulosa cells was determined by qRT-PCR; **(B)** the protein expression level of DLGAP5 in the granulosa cells was determined by western blot; **(C)** the cell viability of granulosa cells was determined by CCK-8 assay; **(D)** the cell proliferation of granulosa cells was determined by EdU assay, scale bar = 100 µm; **(E)** the cell apoptosis of granulosa cells was determined by TUNEL assay, scale bar = 100 µm. *P < 0.05 and **P < 0.01.

LV-DLGAP5 transfection caused a significant increase in the mRNA and protein expression level of DLGAP5 in granulosa cells (**Figures 9A, B**). DLGAP5 overexpression significantly increased the cell viability of granulosa cells (**Figure 9C**). The EdU assay demonstrated that DLGAP5 overexpression significantly increased the number of EdU-positive granulosa cells (**Figure 9D**). Consistently, the TUNEL assay demonstrated that DLGAP5 overexpression markedly decreased the number of TUNEL-positive granulosa cells (**Figure 9E**).

**Figure 10:**
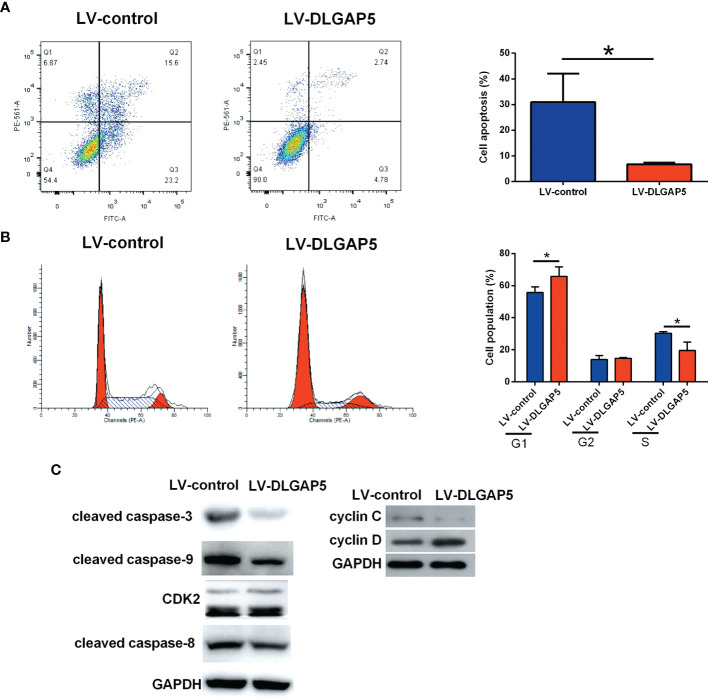
Effects of DLGAP5 overexpression on the granulosa cell apoptosis and cell cycle. Granulosa cells were transfected with LV-control or LV-DLGAP5, and after transfection, **(A)** the cell apoptotic rates and **(B)** cell cycle of granulosa cells were determined by flow cytometry; **(C)** the protein levels of cleaved caspase-3, cleaved caspase-9, CDK2, cleaved caspase-8, cyclin C and cyclin D in granulosa cells were determined by Western blot assay. *P < 0.05.

DLGAP5 overexpression significantly decreased the number of apoptotic granulosa cells (**Figure 10A**). DLGAP5 overexpression caused a significant increase in the cell population of G1 phase and a significant decrease in the S phase but had no effect on the cell population in the G2 phase (**Figure 10B**). DLGAP5 overexpression decreased the protein levels of cleaved caspase-3, -8 (slightly) and -9 and cyclin C, but increased the protein level of CDK-2 and cyclin D in granulosa cells (**Figure 10C**).

## Discussion

The morphological characteristics of PCOS women is the accumulation of follicles of diameter of 2–8 mm, which indicates the selection of the dominant follicle fails and the larger antral follicles have been blocked during development. Usually, antral follicle growth retardation is related to an abnormal endocrine environment, namely, excessive secretion of luteinizing hormone, insulin, or androgen. The net effect is the secondary inhibition of FSH, which inhibits the maturation of other healthy follicles. However, new evidence shows that the inherent abnormal factors of follicular formation in PCOS can affect the early stage of follicular development, manifested as an increase in the density of pre-antral follicles. The follicular dynamics are changed (12). However, the underlying molecular mechanism is uncertain.

In the current study, we used RNA sequencing to provide the lncRNA and mRNA profiles in human ovary granulosa cells from PCOS and healthy ovaries. A total of 1,936 DEGs were identified, and KEGG analysis has some unexpected results. The DEGs were closely linked to olfactory transduction pathways, similar to the study by Jiao (13). Furthermore, AS analysis was performed. Under normal conditions, strict regulation of AS is necessary for the complex tissue function, whereas aberrant splicing appears to an underlying cause for dysfunction and disease, suggesting individual isoforms may serve specific roles (14). In this study, AS analysis displayed no differences between PCOS and control groups regarding the alternative splicing type or gene numbers.

Then, we moved our focus on the DEGs. We validated the expression of AREG, Lin001778, DLGAP5, and PADI6. DLGAP5 was identified as a specifically expressed gene in PCOS. DLGAP5 (also called HURP) is a potential cell cycle regulator that may play a role in the carcinogenesis of cancer cells (15), which has the function of promoting microtubule polymerization and bipolar spindle formation (15). Cells stably transfected with DLGAP5 obtained the characteristics of tumor cells, and silence of this gene could theoretically revert cancer cells to a more normal phenotype (15). For bioinformatic analysis, DLGAP5 was mainly enriched in cell proliferation, mitotic nuclear division and mitotic cell cycle and the pathways involved progesterone-mediated oocyte maturation, p53 signaling pathway, oocyte meiosis and cell cycle (16).

It was demonstrated the proportion of growing follicles increased in PCOS ovaries compared with normal ovaries. In addition, no reduction in the density of primordial follicles was observed in the polycystic ovary compared with normal ovaries (17), and the age of menopause in women with a history of PCOS is similar to that in a control population (18). Therefore, the explanation might be that atresia (loss of follicles) is reduced (i.e., increased survival) during folliculogenesis. In addition, Webber et al. demonstrated the lower rate of follicle atresia during culture in tissue obtained from polycystic ovaries than that observed in biopsies from normal ovarian cortex (19). Apoptosis is the mechanism underlying follicular atresia and is fundamental to the cyclical growth and regression of follicles in the human ovary, and atresia could be caused by granulosa cell apoptosis (20). Throughout oocyte development, granulosa cells provide a suitable microenvironment for follicular development and oocyte maturation and oocyte, promoting growth and differentiation of the granulosa cells (21). Therefore, the dysfunction of these cells or prolonged survival of granulosa cells may contribute to the abnormal folliculogenesis observed in PCOS, which was supported by the study of Das that lower apoptotic rates were found in granulosa cells from patients with PCOS, compared with women with regular ovulatory cycles. In addition, apoptotic inducers and inhibitors were also aberrantly expressed in PCOS group. Cellular inhibitor of apoptosis protein-2 (c-IAP2) was upregulated and caspase-3 positive cells was higher in PCOS group (7).

Usually, PCOS is accompanied with a high LH level. The amplification of LH action on granulosa cells may affect folliculogenesis. High LH may cause inhibition of apoptosis in granulosa cells in PCOS, as LH was reported to prevent cisplatin-induced apoptosis in ovarian cancer or apoptosis in oocytes (22, 23). Under this condition, apoptotic and or proliferation regulator show aberrant expression. In this study, overexpression of DLGAP5 was identified and confirmed. It inhibited apoptosis of granulosa cells in the follicles, which may reduce atresia during folliculogenesis. DLGAP5 knockdown increased the number of apoptotic granulosa cells, which were blocked in the S phase. These may be mainly due to the initiation of caspase 9, followed by executioner caspase 3 activation.

The difficulty in this study was the collection of control samples. Control group includes patients who were diagnosed with endometrial carcinoma or cervical cancer and underwent ovarian biopsy to exclude ovarian lesions. As the sample size was not so big, we cannot subdivide the PCOS patient into different clinical phenotypes and make internal comparisons among the factors, such as high androgen, obesity (BMI ≥25), anovulatory and so on. Still, we need to verify DLGAP5 expression in granulose cells isolated from follicle fluid on a large scale and do some correlation. Finally, the present study was only focused on the DLGAP5, and the interaction between DLGAP5 and the differentially expressed lncRNAs detected in the study may require further examination in future studies.

### Conclusion

Overall, the results of this study showed the different lncRNA and mRNA profile in the granulosa cells from control and PCOS patients. DLGAP5 was identified as a specifically expressed gene in PCOS and its role in the pathogenesis of PCOS may be related to granulosa cell apoptosis.

## Data Availability Statement

The datasets presented in this study can be found in online repositories. The names of the repository/repositories and accession number(s) can be found below: BioSample Accessions SAMN23802955, SAMN23802956, SAMN23802957, SAMN23802958, SAMN23802959, SAMN23802960, SAMN23802961, SAMN23802962, SAMN23802963, SAMN23802964, SAMN23802965, SAMN23802966, SAMN23802967, SAMN23802968, SAMN23802969, SAMN23802970, SAMN23802971, SAMN23802972, SAMN23802973.

## Ethics Statement

The studies involving human participants were reviewed and approved by the Institutional Review Board at the Chinese University of Hong Kong. The patients/participants provided their written informed consent to participate in this study.

## Author Contributions

YD, HL, YS and PWC collected samples and performed experiments. JC, DC, YZ and YS analyzed results. TT, TCL and CCW designed the research strategy and overall experimental plan. YD and YX wrote the original draft of the manuscript and all authors contributed to its revision. All authors listed have made a substantial, direct, and intellectual contribution to the work and approved it for publication.

## Funding

This study was supported by the Health and Medical Research Fund from Food and Health Bureau, The Government of the Hong Kong Special Administrative Region (04152646), the Guangzhou Science and Technology Program Project (202002030173), Panyu District Key Medical and Health Discipline Project (2019-Z04-09) and the National Natural Science Foundation of China (No. 81802551).

## Conflict of Interest

The authors declare that the research was conducted in the absence of any commercial or financial relationships that could be construed as a potential conflict of interest.

## Publisher’s Note

All claims expressed in this article are solely those of the authors and do not necessarily represent those of their affiliated organizations, or those of the publisher, the editors and the reviewers. Any product that may be evaluated in this article, or claim that may be made by its manufacturer, is not guaranteed or endorsed by the publisher.

## References

[1] Chung PW Chan SS Yiu KW Lao TT Chung TK. Menstrual Disorders in a Paediatric and Adolescent Gynaecology Clinic: Patient Presentations and Longitudinal Outcomes. Hong Kong Med J = Xianggang Yi Xue Za Zhi (2011) 17:391–7. 21979477

[2] Ovesen PG Moller N Greisen S Ingerslev HJ. [Polycystic Ovary Syndrome Ii. Endocrinol Metabolism] Ugeskrift laeger (1998) 160:265–9. 9454394

[3] Homburg R. Polycystic Ovary Syndrome - From Gynaecological Curiosity to Multisystem Endocrinopathy. Hum Reprod (Oxford England) (1996) 11:29–39. doi: 10.1093/oxfordjournals.humrep.a019031 8671153

[4] Kong GW Cheung LP Lok IH. Effects of Laparoscopic Ovarian Drilling in Treating Infertile Anovulatory Polycystic Ovarian Syndrome Patients With and Without Metabolic Syndrome. Hong Kong Med J = Xianggang Yi Xue Za Zhi (2011) 17:5–10. 21282820

[5] Norman RJ Dewailly D Legro RS Hickey TE. Polycystic Ovary Syndrome. Lancet (London England) (2007) 370:685–97. doi: 10.1016/S0140-6736(07)61345-2 17720020

[6] Barnes RB Rosenfield RL Namnoum A Layman LC. Effect of Follicle-Stimulating Hormone on Ovarian Androgen Production in a Woman With Isolated Follicle-Stimulating Hormone Deficiency. New Engl J Med (2000) 343:1197–8. doi: 10.1056/NEJM200010193431614 11041762

[7] Das M Djahanbakhch O Hacihanefioglu B Saridogan E Ikram M Ghali L. Granulosa Cell Survival and Proliferation are Altered in Polycystic Ovary Syndrome. J Clin Endocrinol Metab (2008) 93:881–7. doi: 10.1210/jc.2007-1650 PMC2679149 18073308

[8] Homburg R Ray A Bhide P Gudi A Shah A Timms P. The Relationship of Serum Anti-Mullerian Hormone With Polycystic Ovarian Morphology and Polycystic Ovary Syndrome: A Prospective Cohort Study. Hum Reprod (Oxford England) (2013) 28:1077–83. doi: 10.1093/humrep/det015 23377771

[9] Ehrmann DA Barnes RB Rosenfield RL. Polycystic Ovary Syndrome as a Form of Functional Ovarian Hyperandrogenism Due to Dysregulation of Androgen Secretion. Endocrine Rev (1995) 16:322–53. doi: 10.1210/er.16.3.322 7671850

[10] Yurttas P Vitale AM Fitzhenry RJ Cohen-Gould L Wu W Gossen JA. Role for PADI6 and the Cytoplasmic Lattices in Ribosomal Storage in Oocytes and Translational Control in the Early Mouse Embryo. Development (2008) 135:2627–36. doi: 10.1242/dev.016329 PMC2708103 18599511

[11] Huang Y Zhao Y Yu Y Li R Lin S Zhang C. Altered Amphiregulin Expression Induced by Diverse Luteinizing Hormone Receptor Reactivity in Granulosa Cells Affects IVF Outcomes. Reprod Biomed Online (2015) 30:593–601. doi: 10.1016/j.rbmo.2015.03.001 25911599

[12] Franks S Stark J Hardy K. Follicle Dynamics and Anovulation in Polycystic Ovary Syndrome. Hum Reprod Update (2008) 14:367–78. doi: 10.1093/humupd/dmn015 18499708

[13] Jiao J Shi B Wang T Fang Y Cao T Zhou Y. Characterization of Long non-Coding RNA and Messenger RNA Profiles in Follicular Fluid From Mature and Immature Ovarian Follicles of Healthy Women and Women With Polycystic Ovary Syndrome. Hum Reprod (Oxford England) (2018) 33:1735–48. doi: 10.1093/humrep/dey255 30052945

[14] Wang Y Liu J Huang BO Xu YM Li J Huang LF. Mechanism of Alternative Splicing and Its Regulation. Biomed Rep (2015) 3:152–8. doi: 10.3892/br.2014.407 PMC4360811 25798239

[15] Tsou AP Yang CW Huang CY Yu RC Lee YC Chang CW. Identification of a Novel Cell Cycle Regulated Gene, HURP, Overexpressed in Human Hepatocellular Carcinoma. Oncogene (2003) 22:298–307. doi: 10.1038/sj.onc.1206129 12527899

[16] Ke MJ Ji LD Li YX. Bioinformatics Analysis Combined With Experiments to Explore Potential Prognostic Factors for Pancreatic Cancer. Cancer Cell Int (2020) 20:382. doi: 10.1186/s12935-020-01474-7 32782440 PMC7414559

[17] Webber LJ Stubbs S Stark J Trew GH Margara R Hardy K. Formation and Early Development of Follicles in the Polycystic Ovary. Lancet (London England) (2003) 362:1017–21. doi: 10.1016/S0140-6736(03)14410-8 14522531

[18] Wild S Pierpoint T McKeigue P Jacobs H. Cardiovascular Disease in Women With Polycystic Ovary Syndrome at Long-Term Follow-Up: A Retrospective Cohort Study. Clin Endocrinol (2000) 52:595–600. doi: 10.1046/j.1365-2265.2000.01000.x 10792339

[19] Webber LJ Stubbs SA Stark J Margara RA Trew GH Lavery SA. Prolonged Survival in Culture of Preantral Follicles From Polycystic Ovaries. J Clin Endocrinol Metab (2007) 92:1975–8. doi: 10.1210/jc.2006-1422 17341570

[20] Yu YS Sui HS Han ZB Li W Luo MJ Tan JH. Apoptosis in Granulosa Cells During Follicular Atresia: Relationship With Steroids and Insulin-Like Growth Factors. Cell Res (2004) 14:341–6. doi: 10.1038/sj.cr.7290234 15353131

[21] Orisaka M Tajima K Tsang BK Kotsuji F. Oocyte-Granulosa-Theca Cell Interactions During Preantral Follicular Development. J Ovarian Res (2009) 2:9. doi: 10.1186/1757-2215-2-9 19589134 PMC2715405

[22] Rossi V Lispi M Longobardi S Mattei M Di Rella F Salustri A. LH Prevents Cisplatin-Induced Apoptosis in Oocytes and Preserves Female Fertility in Mouse. Cell Death Differentiation (2017) 24:72–82. doi: 10.1038/cdd.2016.97 27689876 PMC5260508

[23] Xia L Wen H Han X Tang J Huang Y. Luteinizing Hormone Inhibits Cisplatin-Induced Apoptosis in Human Epithelial Ovarian Cancer Cells. Oncol Lett (2016) 11:1943–7. doi: 10.3892/ol.2016.4122 PMC4774449 26998105

