# Supplementary figures and images for: Whole Genome Transcriptomic Analysis of Ovary Granulosa Cells Revealed an Anti-Apoptosis Regulatory Gene DLGAP5 in Polycystic Ovary Syndrome
Source: Front Endocrinol (Lausanne). 2022 Mar 18;13:781149. doi: 10.3389/fendo.2022.781149 (PMC8971550; doi:10.3389/fendo.2022.781149)

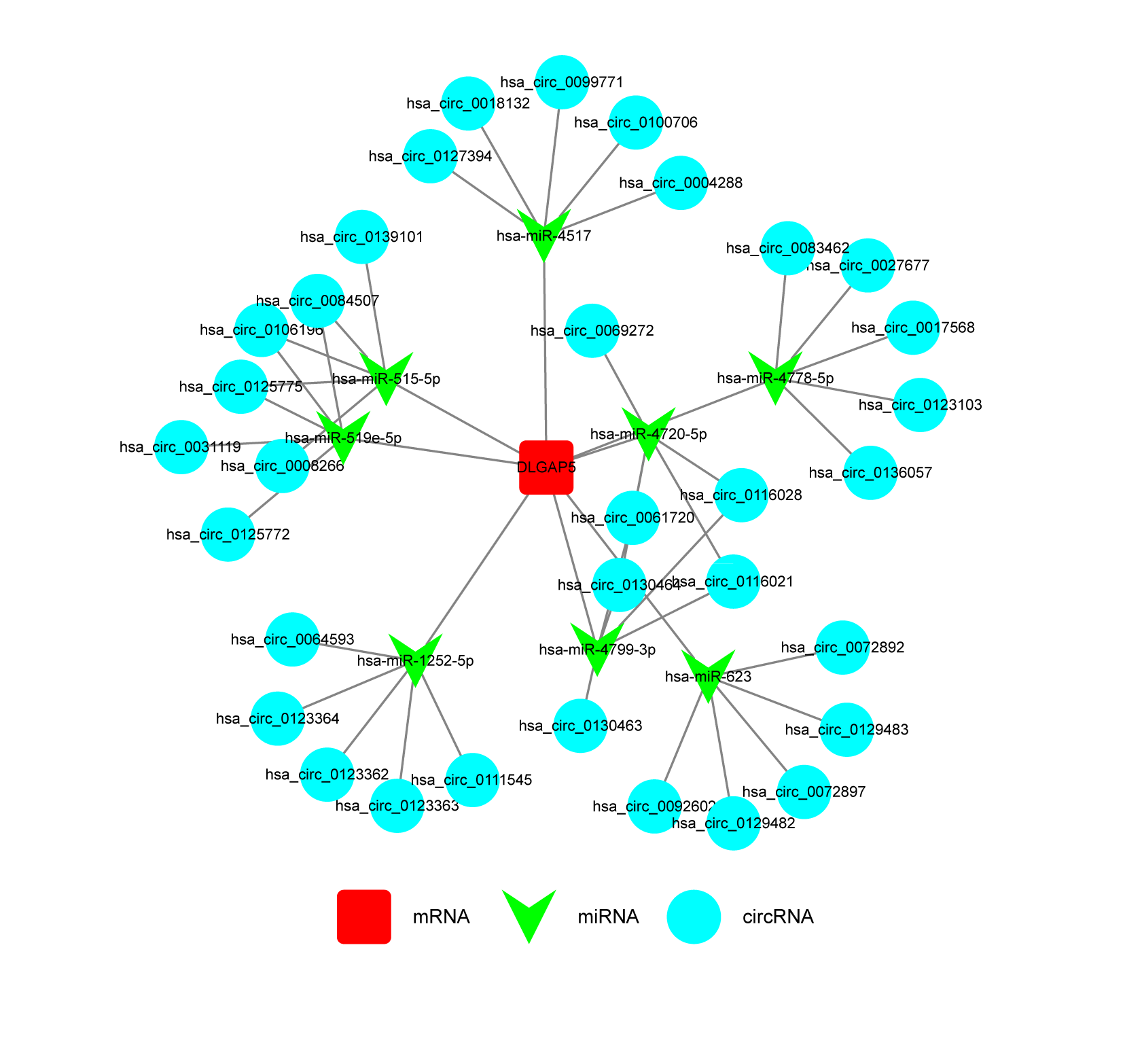

Supplement: Supplementary file 1 [file DataSheet_1.zip › Supplementary Figure S1.TIF]
